# Supplementary material for: Interscapular and Perivascular Brown Adipose Tissue Respond Differently to a Short-Term High-Fat Diet
Source: Nutrients. 2019 May 13;11(5):1065. doi: 10.3390/nu11051065 (PMC6566556; doi:10.3390/nu11051065)

Table S1. Details of probe assays used for qPCR.

Gene	Assay ID
ADRB3 (Thermo)	Rn00565393_m1
ASC1 (Thermo)	qRnoCIP0039017
CITED1 (BioRad)	qRnoCIP0039088
DIO2 (Thermo)	Rn00581867_m1
FGF21 (BioRad)	qRnoCEP0024589
P2RX5 (BioRad)	qRnoCIP0024301
PGC1a (BioRad)	qRnoCIP0022855
SLC36a2 (BioRad)	qRnoCIP0039017
TBX1 (BioRad)	qRnoCIP0027898
TMEM26 (BioRad)	qRnoCEP0028673
NAMPT (BioRad)	qRnoCIP0031455

Table S2. Rat specific Forward and Reverse Oligonucleotide Primers Used for Real-Time PCR.

Gene	Forward primer	Reverse Primer
CIDEA	TCAGTGTCGTATGATATCCGCT	ACCTGGGCAGCATAGGATG
PRDM16	CGAGAAGTTCTGCGTGGATG	GGCACCTTCTTTCACATGCA
UCP1	GCCTAGCAGACATCATCACCT	GTTTCGGCAATCCTTCTGTC
Mtor	TGGAGGGAGAGCGTCTGAGA	TGATGTGCCGAGGCTTTGT

Table S3. Depot fat mass following 72h HFD (grams).

Depot	Chow	HFD
Paracardial AT	0.138±0.012g	0.138±0.023g
iBAT	0.627±0.096g	0.709±0.077g
Perirenal AT	7.48±1.67g	6.98±2.48g
Gonadal AT	6.31±0.83g	5.62±0.38g
Mesenteric AT	4.42±0.94g	5.60±0.71g
Inguinal AT	3.91±0.44g	4.51±0.7g
Total AT	22.87±3.31g	23.51±3.68g

Figure S1. High fat diet (HFD) had no effect 24h energy expenditure (EE) as measured during either the light or dark phases. Data expressed as mean±SEM, n=6 per group.

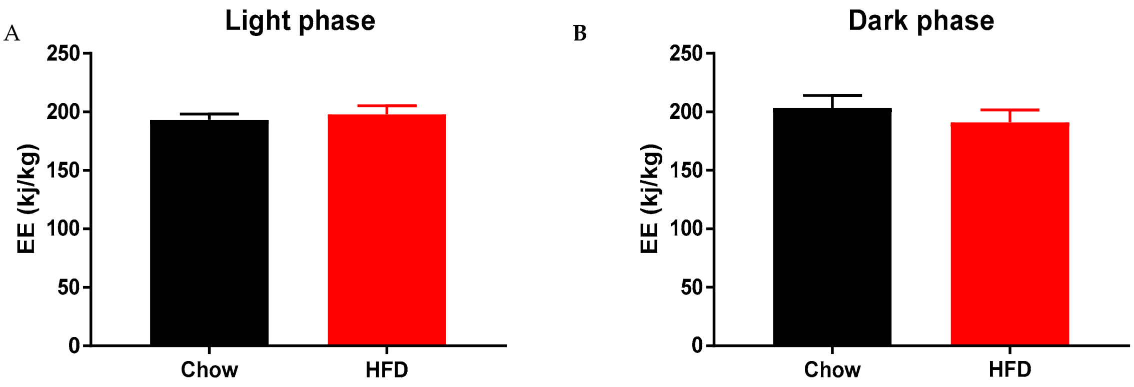

Figure S2. Validation of select genes from targeted array plate. Data expressed as mean±SEM, n=5-6 per group.

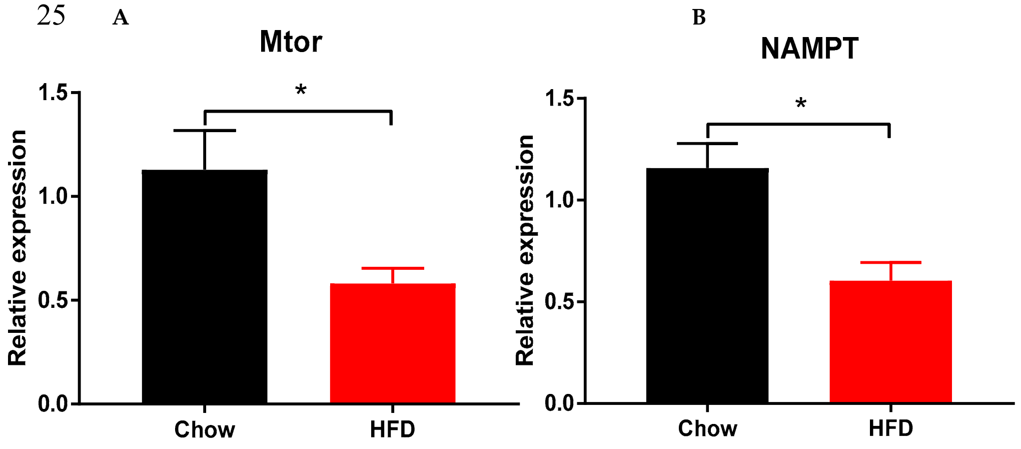

Supplement: Supplementary file 1 [file nutrients-11-01065-s001.pdf]
